# Acknowledgement to Reviewers of *Antibiotics* in 2019

**DOI:** 10.3390/antibiotics9010030

**Published:** 2020-01-15

**Authors:** Antibiotics Editorial Office

**Affiliations:** MDPI, St. Alban-Anlage 66, 4052 Basel, Switzerland

The editorial team greatly appreciates the reviewers who have dedicated their considerable time and expertise to the journal’s rigorous editorial process over the past 12 months, regardless of whether the papers are finally published or not. In 2019, a total of 275 papers were published in the journal, with a median time to first decision of 13 days and a median time from submission to publication of 33 days. The editors would like to express their sincere gratitude to the following reviewers for their generous contribution in 2019:
Abdul-Aziz, HafizLuo, ZonghuaAbia, Akebe Luther KingLupien, AndréanneAbu-Niaaj, LubnaMachowska, AnnaAdiletta, GiuseppinaMacori, GuerrinoAgarwal, SumitMahapatra, Ajit K.Ahern, HollyMahsoub, HassanAkbar, SaminaMahuteau-Betzer, FlorenceAlao, John PatrickMaicas, SergiAlduina, RosaMajtan, JurajAl-Hasan, Majdi N.Makvandi, PooyanAllen, HerbertMallipeddi, Prema LathaAnastasio, AnielloMalwal, Satish R.Ancona-Contreras, VeronicaMan, AdrianAncuceanu, RobertManjunath, ManuboluAndreev, KonstantinMaraolo, Alberto EnricoAndrzejczuk, SylwiaMarc, GabrielAngelini, PaolaMarcone, Giorgia LetiziaAnne, JozefMarotte, HubertAntony, JaneMarti, ElisabetArciello, AngelaMatkowski, AdamArmalyte, JulijaMaxwell, AnthonyArndt, Patrick G.McClelland, Erin E.Arslan, MuhammadMcEwan, NeilArtini, MarcoMcgee, Edo-Abasi U.Asai, DaisukeMelo, LuísAshiru-Oredope, DianeMeneguz, MarcoAskes, SvenMercier, CorinneAugustyniak, AdrianMerda, DéborahAung, Meiji SoeMeškys, RolandasAvent, MinyonMesquita, JoãoBaca, ArthurMIddelveen, MarianneBae, TaeokMilcarek, ChristineBagiu, Iulia-CristinaMillard, AndrewBailey, ConstanceMillemann, YvesBaindara, PiyushMinami, MasaakiBaldin, ClaraMishra, NigamBaldisserotto, AnnaMishra, Sandeep K.Barrott, JaredMiyanaga, AkimasaBeharry, AndrewMohan, Ganesh Babu MalliBeier, RossMonroy, FernandoBekeredjian-Ding, IsabelleMorabito, RosannaBeld, JorisMoreira, CristianaBelitsky, Jason M.Morel, Chantal M.Benito, NatividadMorency-Potvin, PhilippeBerenz, AndrewMori, HirotadaBerghoff, BorkMorita, YujiBernardini, GiuliaMorse, DanielBerthold-Pluta, AnnaMossialos, DimitrisBerti, LilianeMourão, Carlos FernandoBessa, LucindaMukherjee, SomnathBeumer, RijkeltMunteanu, Florentina-DanielaBhattacharya, AnannyaNaber, KurtBhattacharya, SomanonNaka, HiroakiBianco, ArmandodorianoNante, NicolaBilal, MuhammadNath, ArijitBingham, RichardNazki, SalikBisignano, GiuseppeNieto, GemaBlanco-Salas, JoséNimmagadda, AlekhyaBlaskovich, MarkNisnevitch, MarinaBøgwald, JarlNosoudi, NasimBonavia, AnthonyNuñez, María B.Bondon, ArnaudObermüller, NicholasBorchman, DouglasOechslin, FrankBoroviak, KatharinaOgawara, HiroshiBottomley, AmyOgórek, RafałBransfield, RobertOliva, AlessandraBreuker, AnjaOliveira, David DeBroecker, FelixOloso, Nurudeen OlalekanBruchmann, SebastianOneyeaka, HelenBrun, RetoOniga, Smaranda DafinaBrundage, Cord M.Orlando, GiustinoBugarin, AlejandroOves-Costales, DanielBunnell, KristenPaduszynska, MalgorzataBurger, Michael CPal, ChandanBurgos-Ramos, EmmaPang, BoBuyle, FrankyPanzella, LuciaCaffrey, PatrickPapantonopoulos, GeorgeCain, RickyPapini, EmanueleCajal, YolandaPapoutsis, KonstantinosCalcutt, MichaelPapp, JohnCameron, Simon J. S.Papp-Wallace, Krisztina M.Campana, RaffaellaParajuli, SarojCaneiras, CátiaPark, Hyun-WooCarradori, SimonePark, Jun-BeomCarreras, JoaquimParthasarathy, AnutthamanCaruso, GiuliaPascarella, StefanoCasciaro, BrunoPatatanian, EdnaCattò, CristinaPatel, HardikkumarCervero-Aragó, SílviaPaterson, Gavin K.Chamakura, KarthikPathak, AshishChang, Geng-RueiPatini, RomeoChang, Yu-ChaoPatrauchan, Marianna A.Charani, EsmitaPatyra, EwelinaCharvat, RobertPaul, Narayan ChandraChatterjee, JhinukPea, FedericoChattoraj, DhrubaPeabody, DavidChaudhri, VirendraPeczuh, MarkChbib, ChristianePerchec Merien, Anne-MarieChen, ZhaoPeriyasamy, PalsamyCheng, Yuan-BinPidot, SachaChevrot, RomainPikuła, MichałChifiriuc, CarmenPinto, SandraChilders, Delma SusanPiu, SahaChristodoulides, MyronPlonka, PrzemyslawCicciù, MarcoPlotka, MagdalenaCieslar, GrzegorzPoddighe, DimitriClement, CristinaPoirel, LaurentColeman, DavidPokorn, MarkoComans, TracyPope, WelkinConrads, GeorgPorterfield, James ZacharyConsolo, UgoPotter, BethCórdoba, ManuelPozzi, CeciliaCosta-Pinto, Ana RitaPredoi, DanielaCoyne, LucyPrigigallo, Maria IsabellaCriscuolo, ElenaPrincipe, LuigiCushion, Melanie T.Puri, BasantD’Agostino, DonatoQuinto, Emiliano JoséDa Silva, Gabriela JorgeRagusa, AndreaDa Silva, Luis Cláudio NascimentoRahimi, ParvanehDas, SubhaRai, NavneetDavid, MichaelRai, VikrantDe Biase, DanielaRaimondi, Maria ValeriaDe Filippo, DanielaRajamani, SathishDea-Ayuela, María AuxiliadoraRaposio, EdoardoDelorme, VincentRaval, YashDeme, PragneyRehman, Md TabishDenkova-Kostova, RositsaRescigno, AntonioDeplazes, EvelyneResendiz, MarinoDeshpande, GauraviReuter, TimDeslouches, BerthonyReznik, SandraDhakal, DipeshRibeiro, DanielaDi Onofrio, ValeriaRiley, RobertDiana, AlessiaRiska, PaulDíaz Martínez, MargaritaRobben, JohanDifonzo, GrazianaRochani, AnkitDimitrellou, DimitraRodrigues, SophieDing, LingRodríguez-Rojas, AlexandroDinos, GeorgiosRojo, Maria AngelesDixon, NicholasRomero Martínez, ÁngelDkhar, Hedwin KitdorlangRoque, FátimaDoerrler, WilliamRosales-Mayor, EdmundoDolzhenko, Anton V.Rose, KlausDomingo-Calap, PilarRosicka-Kaczmarek, JustynaDomingues, SaraRoy, DipankarDosoky, NouraRubiolo, PatriziaDrlica, KarlRyan, Michael P.Duh, Pin-DerSadowska, BeataDuong, PhuSaelens, JosephDutta, SanjuctaSakamoto, KensakuDziewit, LukaszSalud Bea, Roberto De LaDziuba, DmytroSandanayaka, Atula S. D.Ebata, AyakoSan-Félix, Ana RosaEckroat, ToddSan-Miguel, VeronicaEdin, Matthew L.Santarossa, MaressaEichhorn, IngaSantilio, AngelaElfvin, AndersSaravanakumar, Prof. Dr. KandasamyEllett, FelixSarkar, AmritaElse, Terry AnnSarma, SauravEmbers, MonicaSaturnino, CarmelaErben, MauricioSavage, Paul BErcegović Ražić, SanjaSaxena, VikasEsteve-Romero, JosepSchachter, MichaelFalcó, VicençSchmidt, MartinFaleiro, Maria LeonorSchwan, WilliamFalkinham III, Joseph O.Schweigkofler, WolfgangFallon, BrianSegatto, MarcoFelgueiras, Helena P.Sekiya, MizukiFelicetti, TommasoSelan, LauraFernández, José J.Sellars, JonathanFine, James BurkeSerwer, PhilipFirth, Clair L.Seto, ShigekiFlorindo, Pedro R.Shahi, ShaileshFornaini, CarloShakerley, NicoleFouladkhah, AliyarShang, ZhuoFragkou, Paraskevi C.Shao, QiuyanFranceschini, GiorgioSharkey, LiamFurneri, Pio MariaSharma, IshaGaglione, RosaSharma, ManviGajdács, MárióSharma, MeghaGalanis, AlexisSharma, Narinder KumarGaleazzi, RobertaShetty, AmithGarcia Sanz, JoseShimomura, HirofumiGarcia-Rubio, RocioSierra, Josep M.Gaweł-Bęben, KatarzynaSigusch, Bernd W.Gerard, NormaSilva, Vera L. M.Gerothanassis, Ioannis P.Silvestre, SamuelGervasi, TeresaSimonetti, GiovannaGhadiri, MalihehSingh, PallaviGhosh, ArnabSingh, VineetGhosh, SumitSinha Ray, ShresthaGiacobbe, Daniele RobertoSintim, HermanGiurg, MirosławSiopa, FilipaGodfrey, Henry P.Sistla, SrinivasGolemi-Kotra, DasantilaSivalingam, PeriyasamyGolomb, BeatriceSizochenko, NataliaGomes, Ana P.Skandalis, NikolaosGómez-Salgado, JuanSkowron, KrzysztofGonçalves, Fernando J.Skwor, TroyGonçalves, VirginiaSłomka, ArturGonzález-García, JorgeSmith, Leonard B.Goroll, Allan H.Snyder, LoriGorska-Ponikowska, MagdalenaSoares Silva, Isabel JoaoGrimm, Wolf-DieterSolanki, Manoj KumarGrishin, Alexander V.Sorensen, JohnGruhlke, Martin C. H.Sousa, Sílvia A.Gunia-Krzyżak, AgnieszkaSouza, PedroGuo, JingshuSpiegler, VerenaGupta, CharuSpizzirri, Umile GianfrancoGupta, KajalSplichal, IgorGustafson, John E.Stasiak, MagdalenaHalperin, JohnStatsyuk, Natalia V.Hammer, KatherineStein, DanielHammerl, Jens AndreStȩpnik, KatarzynaHan, QifengStevens, David ColeHansford, KarlStraus, Suzana K.Harada, KazukiStromberg, ZacharyHardefeldt, LauraStubenrauch, ChristopherHarrison, Paul H. M.Stupica, DašaHarrison, SabineSuliman, SaraHayes, Sidney J.Sultana Rekha, RokeyaHe, AinaSummers, James KevinHeney, ClaireSun, KunfengHijano, Diego R.Sundaram, KruthikaHildreth, BlakeSusanne, KaeslerHo, Shih-ChingSutton, J. MarkHodges, TylerSweeney, TimothyHoffman, AngelaSzweda, PiotrHolmes, NeilT. Cenci-Goga, BeniaminoHolt, Scott M.Taglietti, Angelo MariaHong, Seok HoonTan, Bee KHönig, VáclavTang, AihuaHoogland, DominiqueTanini, DamianoHorhat, Florin GeorgeTappa, KarthikHorswill, AlexanderTeng, Ru-JengHousman, Seth T.Teoh, Yu-YaoHu, HelenThanki, Anisha MahendraHu, JunhuiThekkiniath, JoseHubin, TimThomsen, ThomasHunfeld, Peter KlausTinoco, Arthur D.Iebba, ValerioTiwari, Rakesh K.Iijima, NoriakiTjernberg, IvarImperatore, RobertaTkavc, RokIram, SurtajToiu, AncaJacob, MeganToledo, AlvaroJadeja, RavirajsinhToranzo, Alicia E.Jain, PrashantToronto, Coleen E.Jiahuai Hu, AlexTorres Sangiao, EvaJing, RanTrecarichi, Enrico MariaJing, WenwenTrinh, TrangJordao, LuisaTripathi, Jitendra KumarJusto, Julie AnnTroutman, Jerry M.Kachrimanidou, VasilikiTsioutis, ConstantinosKadam, AvinashTsuchida, SachioKahler, CharleneTsukahara, TakamitsuKalatzis, Panos G.Tulkens, PaulKamysz, ElżbietaUlanova, DanaKaneko, JunUmerska, AnitaKang, ChanKyuUpdegrove, Taylor B.Karpiński, Tomasz M.Urban, PhilippeKashiwagi, AkikoUrra, José MiguelKatikou, PanagiotaUsuki, YoshinosukeKato, HirohitoVaira, BernardinoKatsifas, Efstathios A.Vamanu, EmanuelKavita, KumariVan Aerschot, ArthurKenna, Dervla T.D.Vautier, SimonKhaled, YasserVázquez Sánchez, DanielKholmukhamedov, AndalebVelkov, TonyKhurshid, ChrowVenturini, CarolaKhurshid, ZohaibVerni, MichelaKieffer, NicolasViñas, MiguelKim, HoonVisentin, MicheleKim, Jae-SeokVolcho, KonstantinKim, Tae KonVouros, IoannisKimura, Ken-ichiWadsworth, Crista B.King, CarolineWagemans, JeroenKinoshita, NorikoWalker, AndrewKlitgaard, JanneWallace, Joshua S.Ko, Sung-kyunWang, KunKobayashi, NobumichiWang, LeiKöck, RobinWang, WentianKocsis, BelaWang, ZhihanKokkinakis, Emmanouil N.Wardle, JonKomaki, HisayukiWertz, PhilipKovacs, RenatoWessely-Szponder, JoannaKozubowski, LukaszWiedman, GregoryKról-Kilińska, ŻanetaWillcox, MarkKropp, MartinaWilliams, E. MarkKubicova, LenkaWilliams, LisaKubo, MasatakaWilson, PeterKulkarni, ShashankWinders, HanaKumar, ManishWinston, Jenessa A.Kumar, RameshWright, CatherineKumar, SujeetWróblewska, AgnieszkaKumar, SunilWu, AlanKuo, Yen-WenXavier, Nuno M.Kurtti, TimothyXie, RanLabbozzetta, ManuelaYalowich, Jack C.Lai, Pin-KuangYang, InhoLamas, AlexandreYang, KunLampert, Markus L.Yang, LeiLanot, AntoineYano, HajimeLanza, AntonioYaoita, YasunoriLappa, IliadaYazawa, TakashiLarussa, TizianaYero, DanielLaudadio, EmilianoYokota, Shin-ichiLe Brun, Anton P.You, HyunjuLee, Ching-ChiYukl, Erik T.Leitão, Jorge H.Zabetakis, IoannisLenhard, Justin R.Zaharia, CatalinLetek, Michal (Spain)Zapico, Sara CasadoLetek, Michal (UK)Zarfel, Gernot E.Leung, Chung Yin (Joey)Zaric, SvetislavLi, An-DongZeidi, MahdiLi, WenyiZeleny, ReinhardLi, Xian-ZhiZendo, TakeshiLiebman, MichaelŻesławska, EwaLin, Chi-JanZhang, ChenweiLin, RubyZhang, ErshuaiLin, WenZhang, TingLin, Yuh-YihZhang, XinxingLione, Guglielmo GianniZhang, YingLiu, XiaoxiZhong, CuiqingLiu, YanhongZhou, MiLlor, CarlZhu, JieLloyd, VettZhu, JingenLoman, BrettZhu, LuchangLópez, ConcepciónZhu, NinaLopez-Jornet, PiaZhukova, NataliaLorenzi, VanninaZiora, ZytaLorenzo-Gómez, Maria-FernandaŻołek, TeresaLous, JørgenŽuvela, PetarLuedtke, Brandon


